# The Present State of Opinion on Antiseptic Surgery in the United States

**Published:** 1882-05

**Authors:** Edmund Andrews


### Article II.

### The Present State of Opinion on Antiseptic Surgery in the United States. By Edmund Amdrews, m.d.

The main principles of antiseptic surgery have been approved by the majority of American surgeons. Strict Listerism, however, by which I mean, an adherence to all Lister's peculiar methods, is not generally accepted. I have recently instituted some inquiries on the subject in Chicago, Philadelphia, and New York, with the following results: In Chicago, we generally believe in the principles of antisepticism, but we adopt numerous variations in the methods of application, and do not servilely follow Lister's details.

Drs. Isham, Owens, Gunn, Lee, Fenger and myself are probably of substantially the same opinion in this respect.

Mercy Hospital commenced under my direction to use the antiseptics in modern style some seventeen years ago, simultaneously with Grey's Hospital, London. I at first tried Lister's earlier materials, viz: carbolic putty, carbolic oil and tinfoil, but soon found them inconvenient, and substituted methods of my own, which practically worked better, which in modified form I still use. I only employ the spray when cavities are to be opened, not deeming it any better than a fountain douche in simple external wounds.

The County Hospital, for a good while, only used the varying methods of the different members of the staff, but some three or four years ago, commenced a full Listerian system, performing all operations, and even all ward dressings, under the spray.

More recently this has been abandoned, and at present the methods do not materially differ from other hospitals. St. Luke's and the Michael Reese hospitals follow the modified antiseptic customs of Drs. Owens, Schmidt and Park, who attend them.

In Philadelphia, the canvass of votes is nearly as follows: The elder Dr. Gross ridicules the antiseptic system, or at least did so some time ago. Dr. J. Ashurst, Jr., has no disposition to follow Lister, but occasionally uses a wash of permang. potass.

His usual wash for wounds consists of equal parts of alcohol and water, and his cerates contain oxide of zinc or resin.

Dr. Nuncrede is a strict imitator of Lister, but uses parafine paper instead of Macintosh.

Dr. Hunter discards Listerism and dresses with hot laudanum and simple cerate, with parafine paper outside.

Dr. McLellan has a peculiar sponge dressing.

The hospitals in Philadelphia vary, of course, with the opinions of their surgeons. In a general way, their practice is as follows:

The Philadelphia Hospital gives little attention to Lister, though it uses a carbolated oil on lint, one part to 20 or 40. For a wash to wounds, lead water and laudanum are mixed.

The University Hospital washes with alcohol and water, equal parts, or with lead water and laudanum.

The Presbyterian Hospital also uses alcohol and water, or lead water and laudanum.

The Episcopal Hospital employes, sometimes, strict Listerism, and sometimes nothing antiseptic, except hot laudanum.

The spray is rarely used in Philadelphia, except by Dr. Nuncrede and a few others.

In New York the following items show the drift of opinion:

Dr. Mason, of Bellevue Hospital, uses a modified Listerism, without the spray.

Dr. Little, ditto.

Dr. Markoe, ditto.

Dr. Sands uses chloroform as an antiseptic.

Dr. Weir is an absolutely strict Listerian.

Dr. Willard Parker uses antiseptics only moderately.

In the New York Hospital, only one member of the staff uses full Listerism.

In Bellevue Hospital very little Listerism proper is practiced, though antiseptics are more or less used.

In Roosvelt, St. Vincent's and other hospitals the state of things is about the same; in short, there are very few men in New York who are full Listerians, and on the other hand there are very few who do not make a greater or less use of antiseptic dressings.

Finally, the verdict in the United States is this :

1. Antiseptics are of great practical importance in surgery.

2. It is important to observe the antiseptic general principles, but not desirable to follow all of Lister's complicated methods of application.

3. The spray is not desirable, except in dealing with large joints and serous cavities.

